# Trends in the Timing and Magnitude of Ice-Jam Floods in Canada

**DOI:** 10.1038/s41598-018-24057-z

**Published:** 2018-04-11

**Authors:** Prabin Rokaya, Sujata Budhathoki, Karl-Erich Lindenschmidt

**Affiliations:** 1 Global Institute for Water Security, University of Saskatchewan, 11 Innovation Boulevard, Saskatoon, SK S7N 3H5 Canada; 2 School of Environment and Sustainability, University of Saskatchewan, 117 Science Place, Saskatoon, SK S7N 5C8 Canada

## Abstract

Ice-jam floods (IJFs) are important hydrological and hydraulic events in the northern hemisphere that are of major concern for citizens, authorities, insurance companies and government agencies. In recent years, there have been advances in assessing and quantifying climate change impacts on river ice processes, however, an understanding of climate change and regulation impacts on the timing and magnitude of IJFs remains limited. This study presents a global overview of IJF case studies and discusses IJF risks in North America, one of the most IJF prone regions according to literature. Then an assessment of shifts in the timing and magnitude of IJFs in Canada is presented analyzing flow data from 1107 hydrometric stations across Canada for the period from 1903 to 2015. The analyses show clear signals of climate change and regulation impacts in the timing and magnitude of IJFs, particularly in small basins.

## Introduction

Ice-jam floods (IJFs) pose serious threats to riverine communities in the northern hemisphere where almost 60% of the rivers experience significant seasonal effects of river ice^1^. Compared to open water flood events that occur during the ice free season, IJFs can be more severe since, under the same or lower discharge, they can result in two to three times higher water depths than open water floods^2^. The feature that makes them particularly dangerous is their unpredictability of occurrence. IJFs are often sudden and difficult to anticipate which allows little time for the implementation of contingency measures, especially evacuation^3,4^. In the province of New Brunswick in Canada where detailed damage records are available, IJFs account for only one third of flood events, but they result in two thirds of total flood damages^5^.

**Figure 1:**
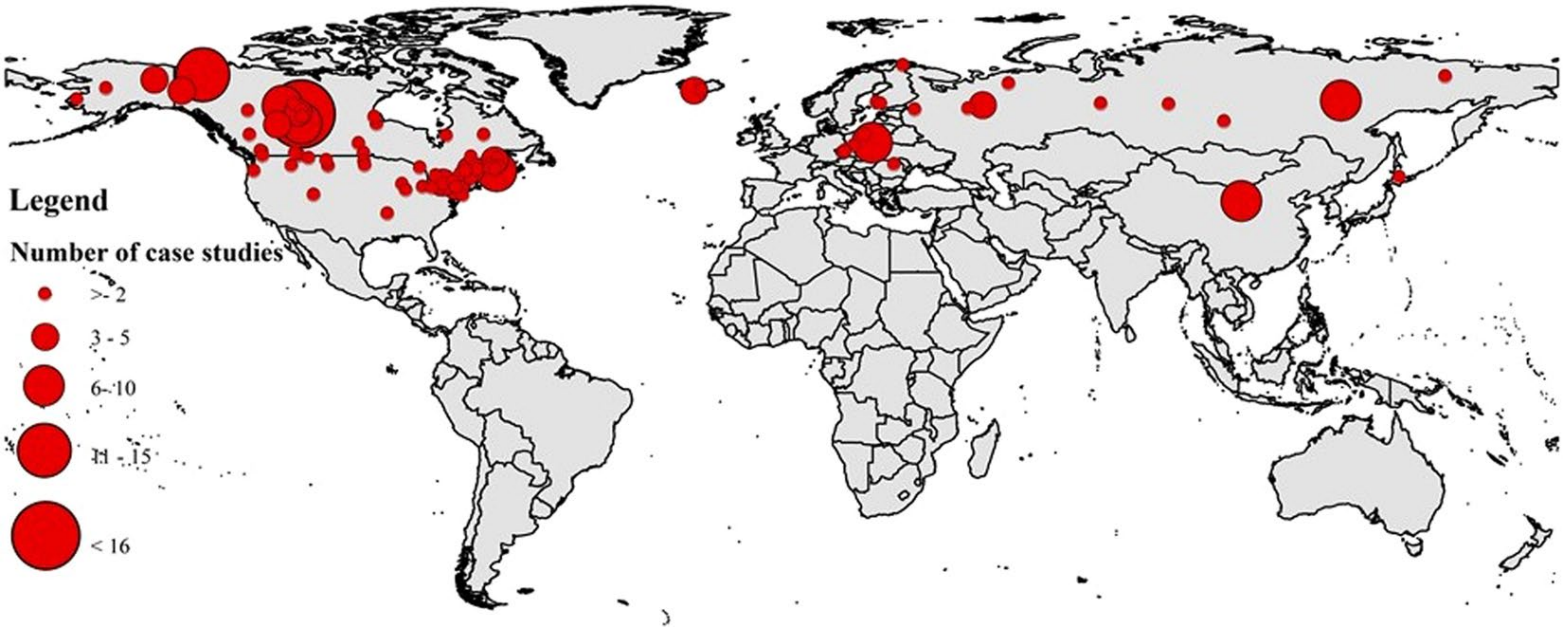
Geographic distribution and occurrence of case studies on IJF. The size of the circle is proportional to the number of case studies reported in the literature.

Figure 1 shows that IJFs are commonly reported in many northern countries across the world. They are largely reported in Canada^6,7^, United States^8,9^, Russia^10,11^, Poland^12,13^ and China^14,15^. It should be noted that the figure is not reflective of IJF susceptible regions in general as it only shows IJF case studies that are reported in journal articles. Many IJF prone areas might not have been reported in journal articles or reported only in conference proceeding papers and/or research reports. Examples are IJFs in Norway and Sweden that are cited in other literature^2,5^ but are missing in Fig. 1. This reveals the gap in reporting in journal articles but not the absence of IJFs in other areas.

The financial costs of IJF events are difficult to estimate and a full economic estimation of damage costs is yet to be conducted^16^. The economic cost of IJFs in North America is estimated to be USD 300 million in 2017^17^. However, these existing approximations could be seriously underestimated considering single IJF events have been known to result in hundreds of millions of dollars in financial damages^1^ such as the 2001 IJF in eastern Russia^18^, the 1996 IJF in Susquehanna River Basin in United States^19^ and the recent IJF event at the interior Alaskan village of Galena in 2013^20^. Moreover, the current estimates account only for tangible monetary costs and do not reflect other serious implications such as residents’ relocation and even loss of life^21^. Table 1 shows the list of the top ten most costly IJFs reported in the literature. Most of these large-scale events in terms of economic losses have occurred in North America.

**Table 1:** Top ten most costly ice-jam floods reported in literature.

SN	Location	Country	Year	Estimated Damage in 2018 (USD)	Source
1	Missouri River	US	1952	1579.42 million	^69^
2	Susquehanna River Basin	US	1996	794.65 million	^19^
3	Irkutsk Region	Russia	2001	281.5 million	^18^
4	Yukon River	US	2013	85.56 million	^20^
5	Town of Peace River in Alberta	Canada	1997	54.03 million	^24^
6	St John River	Canada	1987	45.91 million	^70^
7	Red River	Canada	2009	36.30 million	^71^
8	Yukon River at Eagle	US	2009	33.86 million	^72^
9	New Brunswick and Maine	Canada and US	1991	27.59 million	^73^
10	Lena River	Russia	2010	23.53 million	^74^

**Figure 2:**
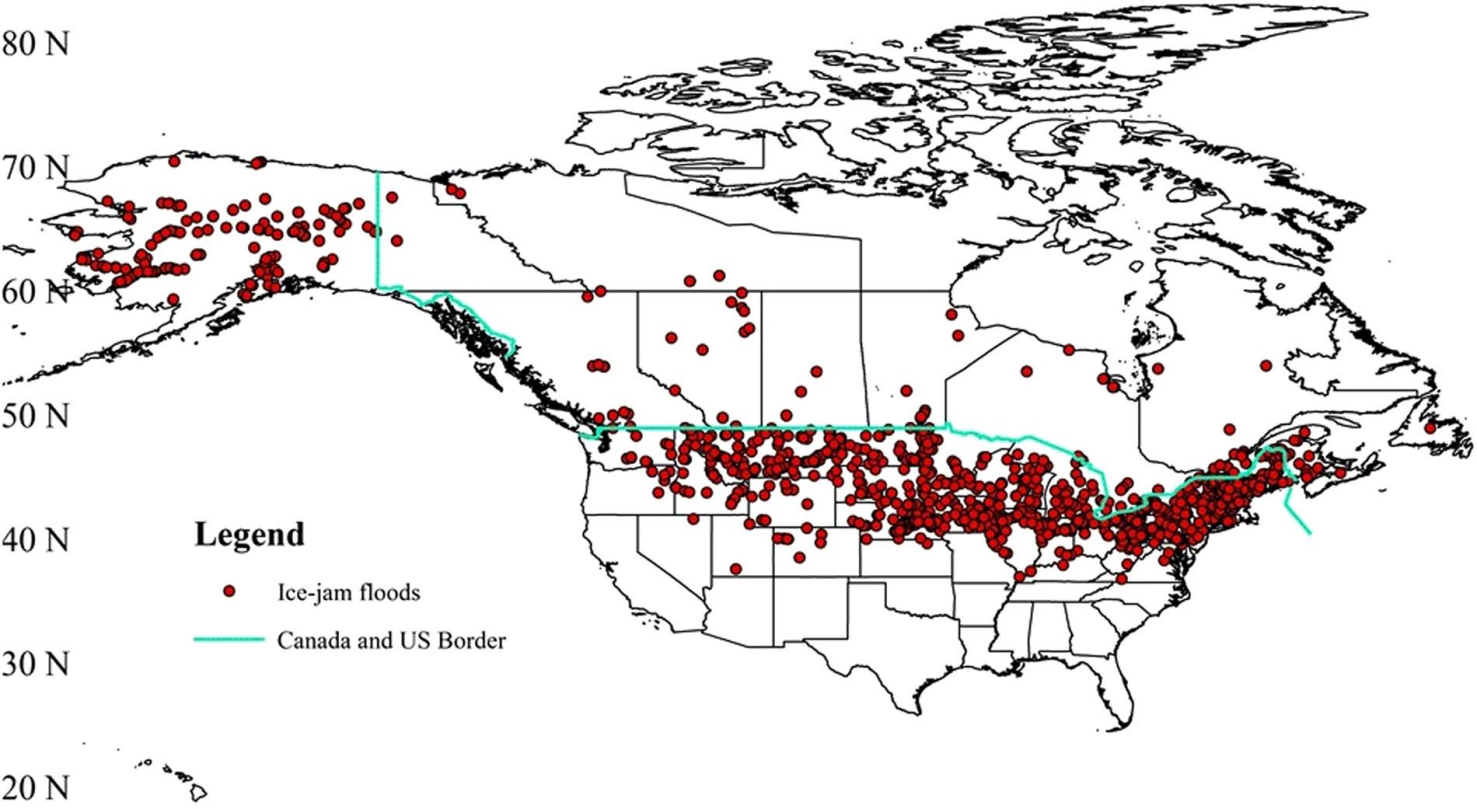
Reported ice-jam floods in North America.

The review of the IJF case studies indicates that North America is one of the most IJF prone regions in the world. Figure 2 shows IJF events that have been reported in the United States and Canada. The sparse IJFs in Canada do not reflect low frequency of IJFs but rather limited availability of IJF records. Nevertheless, the figure does reveal eastern regions to be more prone to IJFs compared to far western regions. Most of the events are also reported between 40–70°N. In the US, the analyses of ice jam data from the Cold Regions Research and Engineering Laboratory (CRREL)^22^ shows that more frequent IJFs are observed in Alaska, New York, Montana, Vermont, Maine, Ohio and Nebraska, each state reporting more than 100 IJF events. Montana and New York are known to be hotspots for ice jamming with each state reporting more than 1400 ice events by 2005^23^. The majority of IJF events featured in the Canadian Disaster Database (CDB)^24^ — 44 out of 83 events — occurred in the provinces of New Brunswick, Ontario and Quebec in east Canada.

With changing climate and continued economic growth, the risk of flood damage is predicted to increase globally^25^. Without any preventive or mitigation measures, the flood damages are estimated to increase up to 20 times by the end of the century^26^. However, increase in global exposure to floods would depend on the degree and characteristics of warming^27^. As high latitude northern regions are more prone to climate warming^28^, the implications of rise in temperature on IJFs might be larger than open water floods^2^. The size of the snowpack and the rate of spring melt controls the severity and magnitude of IJFs in cold regions along with water levels at freeze-up, ice characteristics, meteorological conditions and channel morphology^29–31^. In a warmer world, more precipitation is expected to fall as rain in snow-dominated regions^32^ and the melting of winter snow is to occur earlier in the spring^33,34^ resulting in the shift in timing and magnitude of peak spring runoff^35^.

While all global climate models do not agree in changes in future precipitation, they all point towards increased temperatures^35^. IJFs which occur mainly at the ice cover breakup period in spring are very sensitive to temperature^34^. A warming world, thus, can change the ice cover breakup patterns and result in shifts in timing of IJFs^5^. Strong signals of climate induced shifts in the timing of floods are already observed in continental Europe where northeastern regions are observing earlier spring snowmelt floods whereas earlier winter floods were detected in western Europe^36^. In the northern hemisphere, climatic impacts on ice cover duration have been investigated^37–39^ and regional trends in ice cover freeze-up and breakup have already been reported in the literature^40,41^. However, regional analyses of climate change and anthropogenic impacts on the timing and magnitude of IJFs have not been examined yet, which is a key objective of this study.

In this paper, an assessment of shifts in the timing and magnitude of spring IJFs in Canada is presented. Flow data from 1107 hydrometric stations across Canada for the period from 1903 to 2015 are examined. Theil-Sen slope estimator^42^ is applied to estimate the trend in the timing and magnitude of the IJFs for stations with at least 30 years of data. To quantify the relative impacts of climate change and regulation, we present the results from regulated and unregulated rivers separately. Our analyses show clear spatial patterns of shifts in timing and magnitude of IJFs. We also demonstrate that, compared to large river basins, small basins are more sensitive to changing environment.

## Results

### Shifts in timing of ice-jam floods in Canada

**Figure 3:**
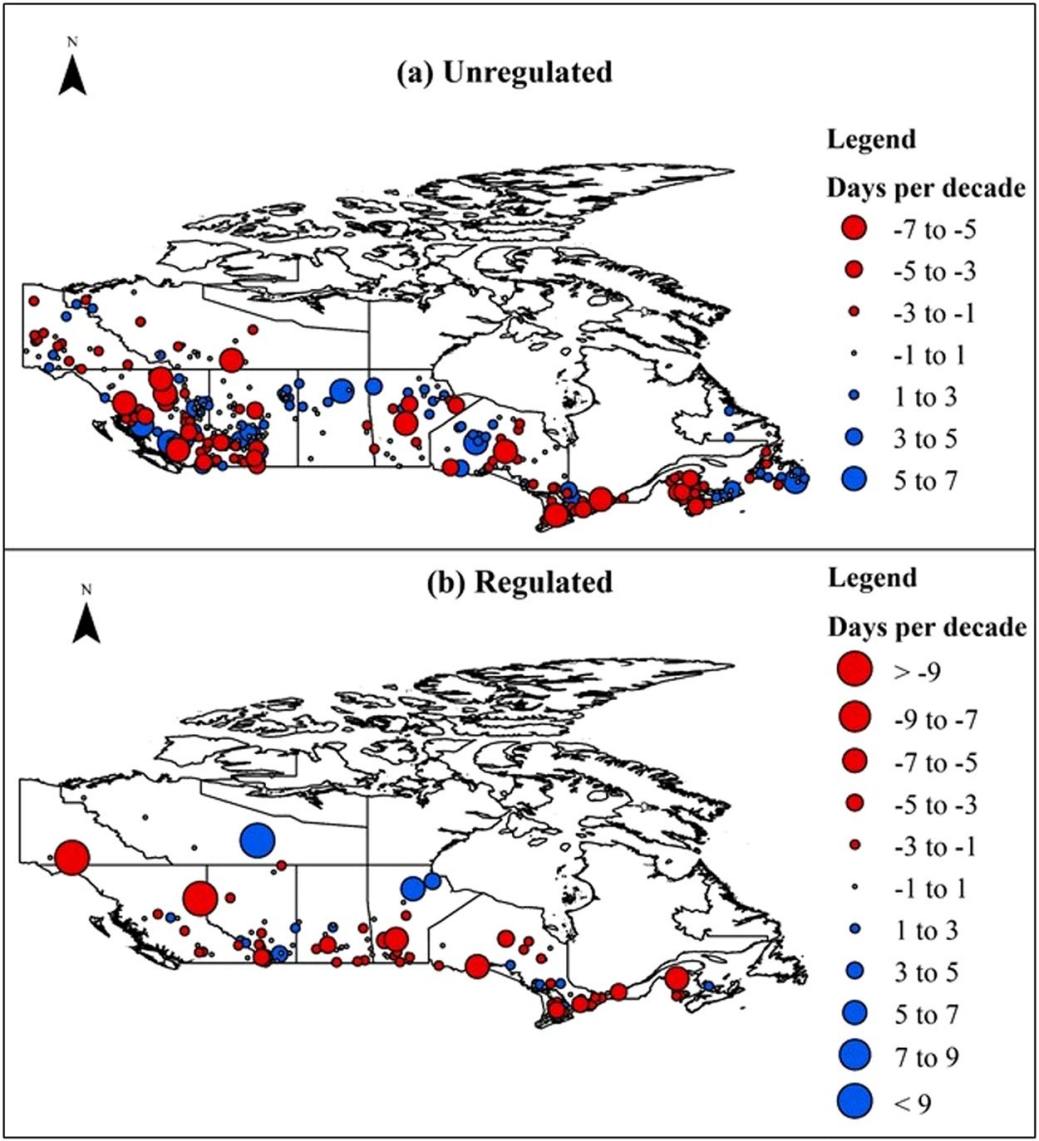
Trends in ice-jam flood timing between 1903 and 2015. The red circles (negative values) indicate early shifts whereas blue circles (positive values) represent delayed shift in timing of IJFs. The size of the circle is proportionate to the magnitude of the shift.

The analyses of the timing of the IJFs show distinct regional patterns. Shifts of one week earlier or later per decade are observed in the timing of IJFs in unregulated rivers and streams (see Fig. 3a). The south-eastern parts and some parts of western Canada show trends towards early IJFs. On the contrary, the central and Atlantic Canada are observed to experience delay in the timing of IJFs. These results are in agreement with findings of other Canadian ice cover breakup studies. As IJFs occur during the breakup period, the results can be compared with previous breakup studies. Similar to our findings, breakup trends of 5 days earlier per century were found in the Yukon River in western Canada^43^ and 11 days per century earlier breakup times were reported for the St. John River in eastern Canada^44^. Likewise, delayed breakup trends in rivers and lakes have been reported in Atlantic Canada^40^, northern Manitoba, Saskatchewan and Northwest Territories^39^. Analyses of breakup trends across Canadian rivers using the Canadian Ice Database also showed similar trends^45^.

Figure 3b shows trends of stations in regulated rivers which are quite similar to unregulated rivers. Some western and eastern parts of Canada show shifts towards earlier IJFs dates whereas some central and northern parts show delays in the timing of IJFs. However, few distinct features distinguish trends in the timing of IJFs between regulated and unregulated rivers. First, the scale of shifts in timing is larger on the regulated rivers (+10 days to −12 days per decade) than on the unregulated ones (±7 days). Second, the regulated rivers in western Canada, especially in the Yukon, British Columbia and Alberta, are observed to experience larger shifts towards earlier IJFs. Globally, regulation has been reported to affect flow regimes in snow-fed rivers more than climate change^46^. And in western Canada, larger contribution of regulation than climate on IJF frequency has been reported^47,48^.

### Shifts in magnitude of ice-jam floods in Canada

**Figure 4:**
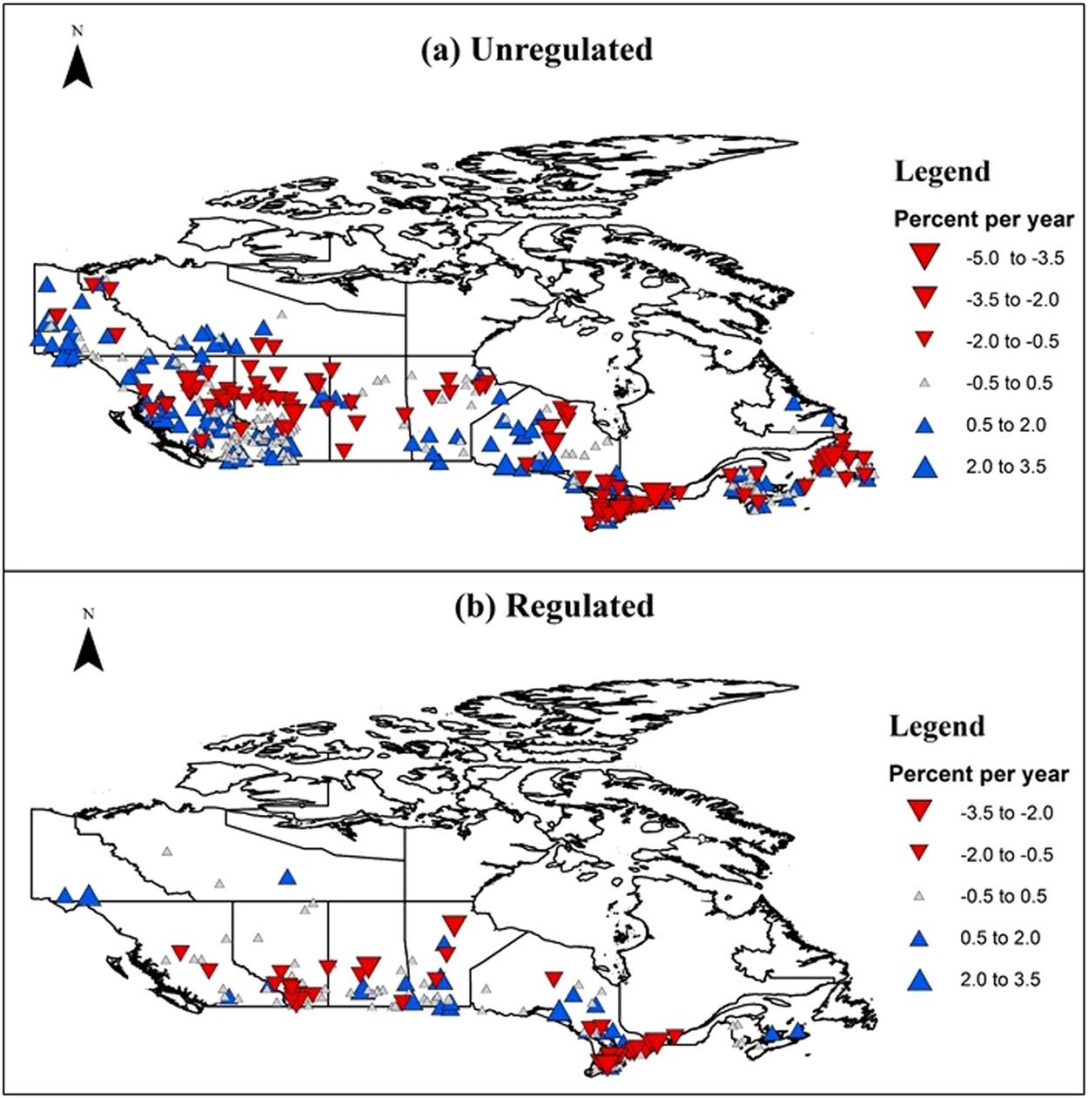
Observed trends of ice-jam flood peak flow from 1903–2015. The red descending triangle (negative values) indicate decrease in magnitude (percentage per year) whereas blue ascending triangle (positive values) represent increase in magnitude (percentage per year) of IJFs. The size of the triangle is proportionate to the magnitude of the trend.

The analyses of the trend in magnitude of IJF peak flow in unregulated rivers also show clear regional patterns. In unregulated rivers and streams, northwest and some central-south regions of Canada are observed to experience increased peak flows while northern Alberta, Atlantic Canada, southern Ontario and northern regions of Saskatchewan and Manitoba show decreasing patterns in magnitude of IJF peak flow (see Fig. 4a). The range of magnitude varies from +3.5% to −5% per year. These results are similar to the findings of other studies on the magnitude of spring floods in Canada. Significant negative trends on spring peak flow in unregulated rivers have been reported in southern Ontario, northern Saskatchewan, Alberta and British Columbia^49^. Another study that analyzed peak April flow for the 1960–1997 period across stations on unregulated rivers in Canada also found similar increasing trends in the magnitude in the Pacific northwest, the South British Columbia Mountains, and the Yukon North British Columbia Mountains^50^. The hydrometric stations in regulated rivers show similar trends with some distinct differences in south western regions (see Fig. 4b). While some areas showed increasing trends in unregulated rivers, regulated rivers show only decreasing trends. Moreover, the range of magnitude in regulated rivers varies from +3.5% to −3.5% per year which is a slightly narrower range than for unregulated rivers which reveal a −5% per year decreasing trend in magnitude of IJFs.

### Sensitivity of the river basins

**Figure 5:**
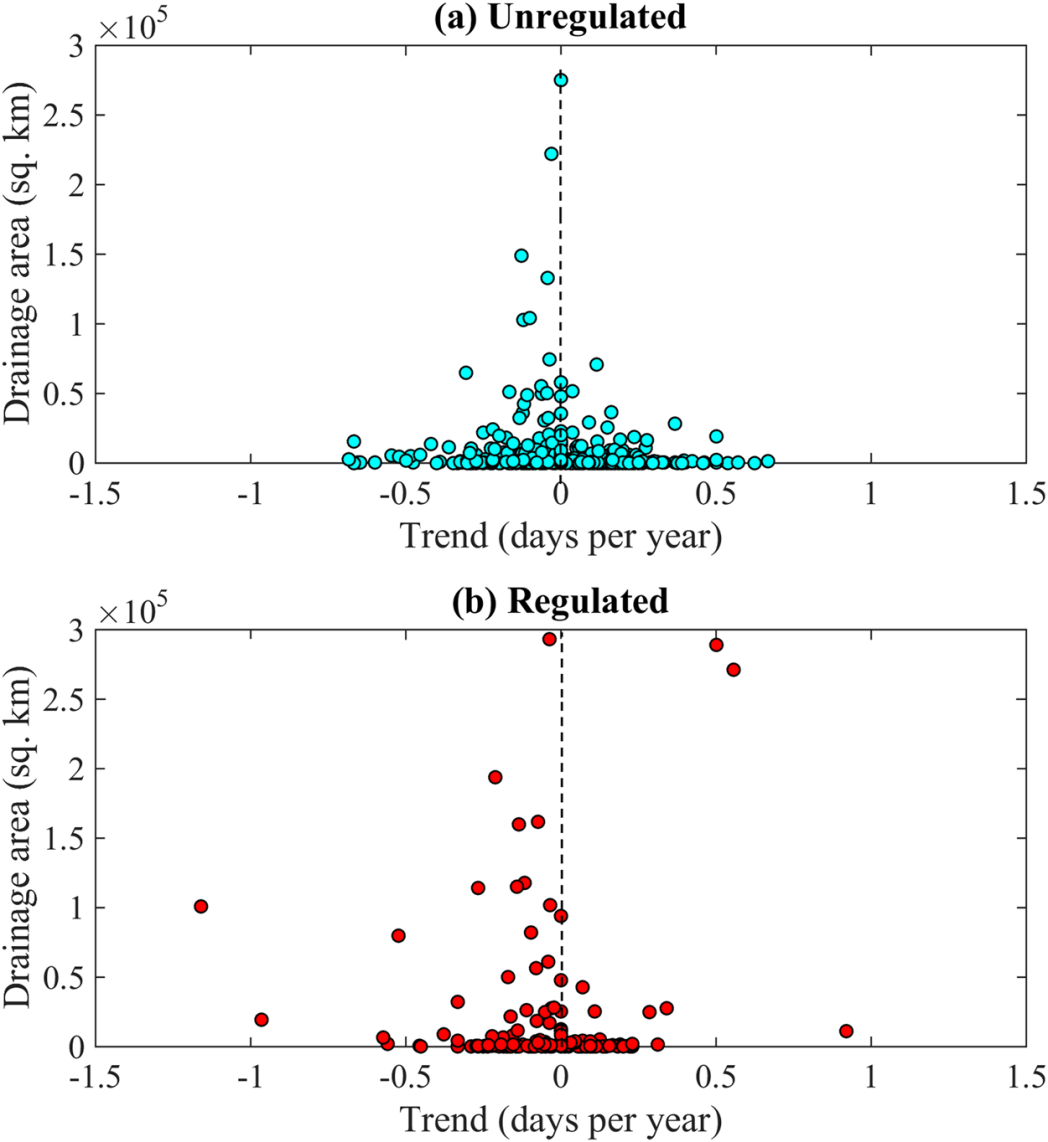
Trends in the timing of IJF plotted against drainage area of the basin. For comparability and clarity, regulated hydrometric stations draining up to 300,000 square kilometers are only shown in this figure.

In the context of a changing climate, small river basins are observed to be more sensitive to changing environment than larger river basins. Figure 5a shows the drainage area of the basin plotted against the slope in timing of IJFs. It reveals that as the size of the basin increases, sensitivity of the system decreases. Thus, small basins show larger shifts which gradually decrease as the size of the basin increases. However, the same cannot be concluded for the regulated basins whose streamflows are more governed by anthropogenic factors. Figure 5b shows large shifts even in larger basins in regulated rivers.

## Discussion

The analyses of case studies show that IJFs are prevalent in most of the northern countries. However, there is a large contribution from North America. Note that this assessment does not indicate the dominance of a specific geographic region in advancing IJF research. It is possible that a large wealth of knowledge on IJF research exists that is written in other languages but is not reflected in this assessment. However, a large proportion of contribution of publications from a specific geographic location may reduce the diversity of issues in IJF research literature. Current river ice knowledge is largely based on large rivers such as those in Canada and Russia compared to relatively small and fairly steep rivers such as those in Norway^51,52^. Thus, the progress and challenges in IJF research across those least represented countries may not be reflected in current literature, a key knowledge base for academics and practitioners.

This assessment also shows that a large number of IJFs occur in the US and Canada. Data show eastern regions to be more prone to IJFs compared to western regions. In particular, US states Alaska, New York, Montana, Vermont, Maine, Ohio and Nebraska report comparatively larger number of IJF events with each state reporting more than 100 IJF cases. In Canada, eastern provinces, particularly New Brunswick, Ontario and Quebec, report more than half of the total IJFs registered in the CDB. In recent years, with increasing air temperatures, mid-winter breakup events have also been reported in temperate and maritime regions of North America, and typically occur during January and February^53^. They are triggered by winter temperatures rising above freezing and/or by rain-on-snow events^54^. Mid-winter breakup events can also result in IJFs and can be more destructive than spring events as they are sudden and difficult to anticipate^44^. They have been reported in Michigan^23^, Maine^55^, Wisconsin^56^ and Alaska^57^ among others in the US and in New Brunswick^44^, Quebec^58^, British Columbia^48^ and Yukon^57,59^ among others in Canada.

The analyses of shifts in timing and magnitude of the IJFs show clear spatial patterns. Western and south-eastern Canada show higher sensitivity. Many of the shifts and trends in unregulated rivers reported in this study can be explained by similar climatic patterns. Previous studies have found the timing of the breakup to strongly correlate with air temperature^60^ and 0 °C isotherms^45^. Significant trends towards earlier springs over most of western Canada with eastern Canada experiencing later springs have been reported^61^. Other studies have also found similar results in spatial and temporal patterns between 0 °C isotherm dates and break-up dates^39^. Warmer spring temperatures have been reported to result earlier snowmelt in Canada^62^ and the timing of the flood closely follows that of snowmelt in snow-dominated regions^36^. Dynamic (mechanical) breakup is more probable in the case of breakup occurring earlier in the season, whereas thermal breakup is more dominant when breakup is delayed allowing ice covers decay due to increasing temperatures and longer exposure to solar radiation^48,63^. While mechanical breakup can cause extreme IJF events, thermal breakup usually does not result in significant jamming^54^. Thus, the regions that show early trends in the timing of IJFs might be more prone to IJFs than those regions showing delayed trends.

If the same trend continues then IJF risks might increase in unregulated rivers in western Canada as results show increasing trends in magnitude and earlier trends in the timing of IJFs. However, it will be difficult to anticipate future trends in regulated rivers. Previous studies have found that breakup flows play a significant role in ice-jam flooding^2,48,63–65^ which in regulated rivers are controlled by human water and energy requirements. Thus, regulation also offers a possibility of reducing IJF risks in regulated rivers. However, though peak spring flow can be modulated, hydropower based regulation will still increase the risks of IJF during the freeze-up period. Larger flows are released during winter months when energy demands are higher, which can result in a higher freeze-up stage in a river. Further, depending upon hydro-meteorological conditions, the initial ice cover may go through consolidation events, i.e. collapse and thickening of a newly formed ice cover, that might result in significantly higher water levels posing serious flood risks to riverine communities^66^. An example includes basement flooding events in the Town of Peace River in Alberta, Canada in 1982, 1992, 2005 and 2008 since regulation in headwaters of the Peace River in 1972 (http://cripe.ca/docs/proceedings/19/Jasek-et-al-2017.pdf).

## Conclusion

IJFs result in significant economic losses in the northern communities. Single IJF events are known to result in hundreds of millions of dollars in damages. An assessment of the global IJF case studies shows that North America is one of the most IJF prone regions in the world. Northeastern US and southeastern Canada were found to be particularly susceptible to IJFs. In recent years, mid-winter breakup events have also been increasingly reported in temperate and maritime regions of North America. In Canada, trend analyses showed distinct increasing and decreasing patterns in the timing of IJFs, with south-eastern and western Canada trending toward earlier IJFs dates whereas Atlantic Canada is experiencing delayed timing in IJF events. While trend patterns are similar between regulated and unregulated rivers, larger implications are observed in regulated rivers with larger shifts in trends. The trends in IJF are also found to be correlated with size of the basins in unregulated rivers with small basins showing larger variabilities and larger basins showing less sensitivity. However, anthropogenic factors influenced regulated rivers did not show similar sensitivity in larger basins.

## Methods

Case studies of IJFs were identified from the literature review analyzing data from the Web of Science. All journal articles published up to October 2017 were identified using the search string (TITLE-ABS-KEY (ice jam* flood*) which resulted in 326 papers. Filtering only journal articles and reading the abstract of all articles yielded only 188 IJF related papers. And a total of 94 case locations were found to have been reported in the 188 articles.

The estimated damage values for 2018 for Canadian IJF events were calculated using the Bank of Canada’s Inflation Calculator (https://www.bankofcanada.ca/rates/related/inflation-calculator/) and for other IJF events using Inflation Calculator of the US Bureau of Labor Statistics (https://www.bls.gov/data/inflation_calculator.htm). The damage values for Canadian IJF events were converted from CAD to USD using Currency Converter of the Bank of Canada (https://www.bankofcanada.ca/rates/exchange/currency-converter/) using the average exchange rate (i.e. 1 CAD = 0.7787 USD) for last one year (Feb 2017–Feb 2018).

IJF data for the continental United States were retrieved from the ‘Ice Jam Database’ of the CRREL of US Army Corps of Engineer. The CRREL data spans from 1780 to the present that provides a record of jams and details of the events^22^. As of October 26, 2017, 22692 ice-jam events have been recorded. When these ice-jam events were filtered for IJFs from the available information on each event description, about 10% of the events, i.e. 2356 cases of IJFs were obtained. Canada, unlike US, does not have an explicit ice-jam database. However, the Canadian Disaster Database (CDB)^24^ records hydrological-meteorological flood events. But, it is a broad category and sub-categories such as rain, snowmelt or ice-jam floods are not available. Moreover, only the events that meet one or more criteria to qualify as ‘disaster’ are recorded (see https://www.publicsafety.gc.ca/cnt/rsrcs/cndn-dsstr-dtbs/index-en.aspx for the list of criteria) which means a large number of localized or small-scale ice-jam flood events are not recorded. A total of 315 hydrological-meteorological flood events were reported. From the list, the summer floods were filtered out and the remaining events were further validated by reading the description provided with each event as well as cross-referencing online information which resulted in 83 large IJF events.

**Figure 6:**
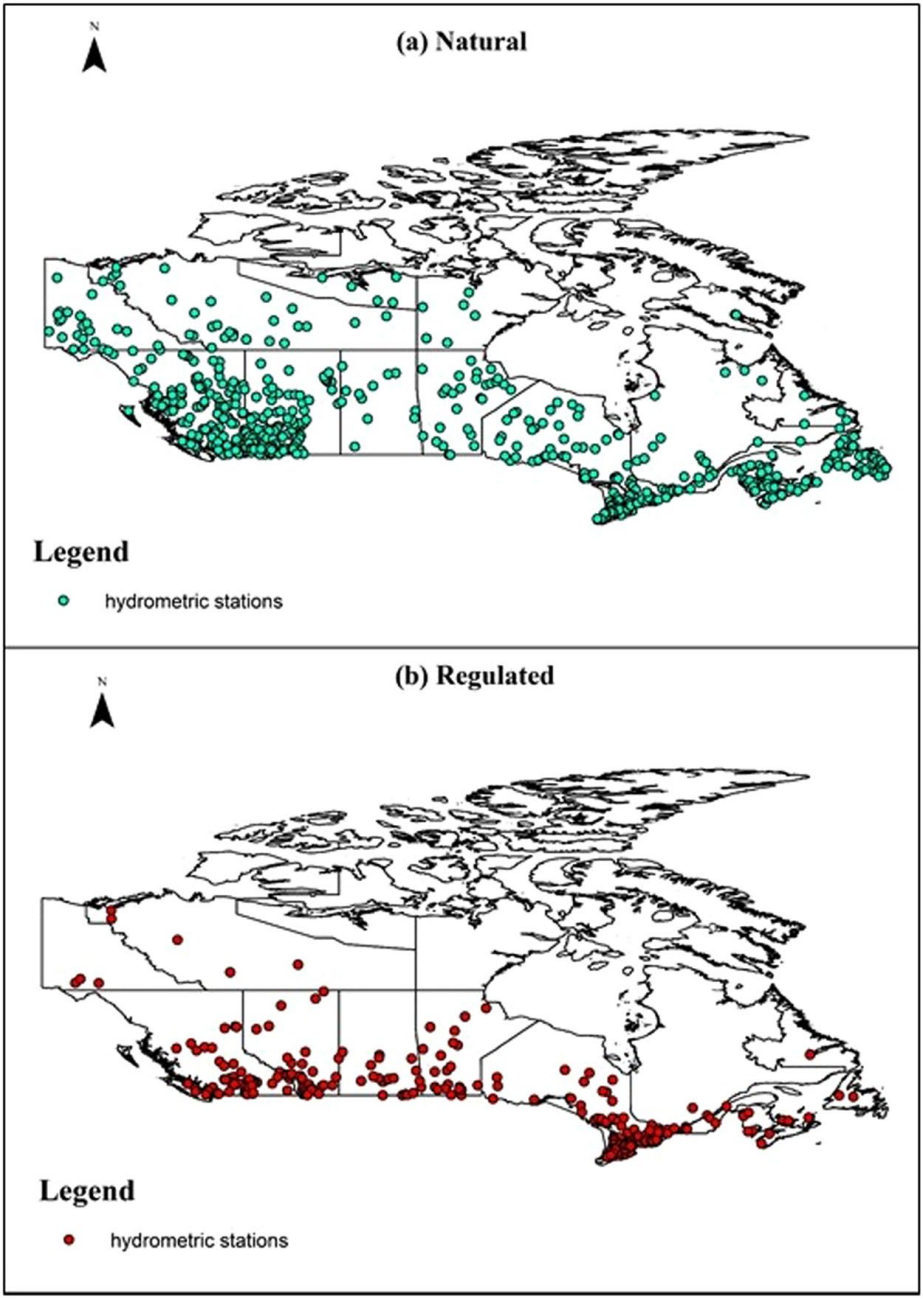
Hydrometric stations used in the study: (**a**) stations in unregulated rivers and (**b**) stations in regulated rivers.

For the analysis of shifts in the timing and magnitude of IJFs, we collected data from hydrometric stations in Canada available from the Water Survey of Canada (https://www.canada.ca/en/environment-climate-change/services/water-overview/quantity/monitoring/survey.html). A total of 7781 stations are available across the country, out of which only 1107 stations are active and have continuous flow measurements for at least 30 years. See Fig. 6 for the locations of stations used in this study. IJF was considered to be the largest peak daily discharge in each calendar year within ±3 days of the last ‘B’ flag of the observed record for each station. The ‘B’ flag, which is provided by the Water Survey of Canada along with hydrometric data, denotes ice-induced ‘backwater’ effects due to the presence of ice at or immediately downstream of the gauge in the river. Thus, the discharge with the first ‘B’ flag indicates the beginning of the freeze-up of the river whereas the last ‘B’ flag is indicative of the end of the ice cover season. This method of using the ‘B’ flag has been previously used to identify freeze-up and breakup dates^40,41^. The ‘B’ flag was further assessed to ensure that backwater conditions resulted from known ice timing effects and not from log-jam or beaver-dam effects^67,68^. The Theil-Sen slope estimator^42^ was then applied to estimate the trend in the timing and magnitude of the floods for stations with at least 30 years of data. This non-parametric estimator was chosen for its robustness and insensitivity to missing values and outliers^36,45^. The trend estimator is the median of the difference of dates over all possible pairs of years within the time series.
